# Symbionts of Red King Crab from the Sea of Okhotsk: A Review of Russian Studies

**DOI:** 10.3390/biology14020148

**Published:** 2025-01-31

**Authors:** Alexander G. Dvoretsky, Vladimir G. Dvoretsky

**Affiliations:** Murmansk Marine Biological Institute of the Russian Academy of Sciences (MMBI RAS), Murmansk 183038, Russia

**Keywords:** *Paralithodes camtschaticus*, red king crab, Sea of Okhotsk, biological aspects, fisheries, symbionts, parasites

## Abstract

The red king crab, *Paralithodes camtscaticus*, is a commercially significant crustacean that supports lucrative fisheries in Russia, the USA, and Norway. The western Kamchatka shelf, located in the Sea of Okhotsk, is home to one of the most important populations of the red king crab. In this study, we have conducted a review of the symbionts associated with *P. camtscaticus* in the waters off the Kamchatka Peninsula. A total of 42 symbiotic species belonging to 14 different phyla were identified in association with the red king crab. Out of these, 14 species were found to be parasitic to the red king crab, while the remaining 28 were either commensal or epibiont in nature. The taxa with the highest number of associated species included ciliates (11), crustaceans (8), and acanthocephalans (4). Our study found that red king crabs suffering from shell disease exhibited a more diverse symbiotic fauna and higher infestation indices as compared to healthy crabs, which were found to be free from parasites. Dangerous symbionts, such as dinoflagellates *Hematodinium* sp. and rhizocephalan barnacles *Briarosaccus callosus*, had low incidence rates, indicating that the red king crab population in the Sea of Okhotsk is in good condition with respect to population abundance, health, and recruitment and is not being adversely impacted by symbiotic organisms.

## Simple Summary

Red king crab is one of the most important and well-known commercial decapods supporting viable fisheries both in their native area, the Pacific Ocean, and in their new habitat, the Barents Sea. Among all red king crab populations, the population existing in the Sea of Okhotsk is the most abundant. Here, we reviewed the main biological aspects, fishery, and symbionts of this species in this region. The symbiotic fauna is quite diverse and composed of commensals, epibionts, and parasites. The latter group includes dinoflagellates and rhizocephalan barnacles, but their infection indices are low and these symbionts seem to have a negligible impact on the population.

## 1. Introduction

The red king crab, *Paralithodes camtschaticus*, is a widely distributed and well-known species [1]. With a leg span that can reach up to 180 cm from tip to tip, it is also the largest of the king crabs [2]. The maximum recorded size of a male red king crab has been measured to be 227 mm in carapace length and 283 mm in carapace width (CW, the greatest straight-line distance across the carapace excluding spines). In comparison, the maximum size for a female was found to be 195 mm in carapace length and 213 mm in carapace width [3]. Red king crab meat is renowned for its high quality in terms of chemical composition, making it a delicacy for subsistence consumption [4]. Furthermore, the current demand for red king crab in world markets is expanding, which has led to an increase in competitively higher prices [5]. Red king crab by-products have been found to contain high concentrations of valuable biomolecules, including fatty acids and enzymes. These by-products have thus been identified as promising candidates for deep processing, a process that yields products with added value, such as chitosan [6,7,8,9,10,11,12,13].

It is possible to identify three large regional groups of *P. camtschaticus* in the North Pacific. The southernmost group can be found in the Gulf of Alaska, where this species exists throughout the fjords and channels of British Columbia and Southeast Alaska. Population density is highest around the archipelago of Kodiak Island [2]. The second geographic group of red king crab is located along the shelf of the Eastern Bering Sea and among the bays and islands on the south side of the Alaska Peninsula. At lower densities, the species can also be found in the bays of the easternmost Aleutian Islands, including Unalaska Island. The highest stock indices are registered on the broad, shallow continental shelf, where the crabs range from Unimak Pass and the Port Moller region north to the Kvichak River in Bristol Bay [2]. The third group, belonging to the West Bering Sea Large Marine Ecosystem, is distributed north from Norton Sound into the Chukchi Sea, southwest along the coast of the Kamchatka Peninsula into the Sea of Okhotsk, and southward along the Kuril Islands to Hokkaido [2]. In Asian waters, seven sub-populations of *P. camtschaticus* are found, among which the West Kamchatka sub-population is the most abundant [5]. In the Atlantic Ocean, a non-indigenous red king crab population occurs in the Barents Sea, following the successful intentional introduction conducted in the USSR in the 1960s [5,14,15]. Fluctuations in red king crab stocks are determined by environmental factors [16,17,18,19,20,21] and fishing pressure [1,14,15,22,23].

It has become widely recognized and acknowledged that numerous marine organisms, including king crabs, can harbor a plethora of symbionts, such as pathogens, parasites, and various other associated fauna [24,25,26,27]. The term “symbiosis” signifies “living together” and encompasses pathogenesis, parasitism, commensalism, mutualism, and phoresy. Consequently, symbionts are organisms that engage in some form of intimate or close association with their respective hosts [28,29,30]. A disease manifests as a dysfunction or an abnormal imbalance within the host [31,32,33]. Both parasites and pathogens can induce disease by disrupting the host organism’s physiological functions [34,35]. Facultative symbionts do not rely on their host physiologically but can establish an opportunistic relationship under specific circumstances. In contrast, obligate symbionts exhibit a physiological dependency on their host. Commensalism, along with its related associations, inquilinism and phoresy, are interactions whereby the symbiont derives benefits while the host remains unaffected [36,37]. Mutualism occurs when both the host and the symbiont reap benefits or derive metabolic dependency from their association [38,39,40].

Symbionts may exert a direct or indirect impact on the health, brood success, survival, and population growth of crabs [38,41,42]. The symbiotic associations involving red king crab have been extensively investigated across its distribution areas in both the Pacific Ocean and the Barents Sea [3,43,44,45,46,47,48,49,50,51]. Furthermore, the relationship between disease, parasitic or otherwise, and the ecology of the natural red king crab population in the Sea of Okhotsk has been well documented [52,53,54,55,56,57,58,59,60,61,62]; however, the majority of these studies were published in Russian, limiting their accessibility to a broader international audience.

In light of this, we present a concise overview of the primary biological aspects of red king crabs from the Sea of Okhotsk, an analysis of fishery dynamics, a survey of organisms that utilize red king crabs as hosts, and an emphasis on essential features of their associations.

## 2. Biological Aspects of Red King Crab in the Sea of Okhotsk

During winter, red king crabs are found at depths exceeding 100 m. Spawning occurs in spring (May–June) at shallower depths (<50 m, sometimes as shallow as 10 m). Male mass molting takes place post-mating in summer [63]. Upon hatching, larvae drift with currents along the western Kamchatka coastline northward toward the Khairyuzovskiy Area and the Northern Closed Area [64], where post-larval settlement and juvenile growth predominantly transpire. The most favorable settlement sites can be found in this region, featuring hard ground, pebbles, and stones along with complex biogenic habitats, including hydrozoans, bryozoans, and macroalgae. Consequently, northern areas function as nursery grounds for the entire metapopulation in the eastern part of the Sea of Okhotsk. Ontogenetic migrations head southward, moving against prevailing currents [63]. Southern area recruitment originates from the north. Seasonal migrations involve red king crabs transitioning between deep water (100–250 m) during winter months to shallow water (4–50 m) for reproduction, and then to medium depths for feeding [5].

Typically, high crab density, an abundance of females, and relatively small mean male sizes—attributable to the high proportion of young and sublegal males (CW < 150 mm)—characterize the northern regions of western Kamchatka. In contrast, southern sub-populations exhibit relatively low crab densities and larger mean male sizes compared to northern areas [65,66]. In waters off the west Kamchatka coast, Mollusca (primarily the bivalves *Siliqua media*, *Tellina lutea*, and *Mytilus* sp.) and Crustacea (dominated by *Balanus* sp. barnacles) represent the predominant food groups for red king crabs, in terms of both wet weight percentage and frequency of occurrence, followed by fish as the next most important prey group [67,68,69].

Male crabs have the capacity to copulate with up to seven females within a single spawning season; however, the fertilization rate is significantly reduced after the first two or three copulations [70]. Moreover, the fertilization rate is also impacted by size, with larger male crabs exhibiting greater success. The average individual fecundity of female red king crabs increases from 60,000 eggs in northern regions to 220,000 in southern areas of the shelf [71].

In male crabs, molting predominantly occurs immediately after the mating season, particularly during the first half of summer [64,72]. This process commences earlier in southern regions, with the area of intensive molting gradually shifting northward. The Ozernovskiy Area is distinct in terms of molting behavior, as some crabs molt during the winter season and do not molt in the subsequent summer. Winter molting has also been recorded in northern areas [72].

The size at which female red king crabs reach sexual maturity is 85–95 mm CW [73,74]. In males, spermatozoa are observed in crabs as small as 80 mm CW [72]. The growth rate of crabs off western Kamchatka is slow: an 8-year-old crab measures 80 mm, a 10-year-old specimen measures 100 mm, a 13-year-old crab measures 135 mm, and a 14- to 15-year-old crab measures 145–155 mm CW. Males measuring 80–130 mm CW are already able to mate [72].

## 3. Fishery

**Figure 1:**
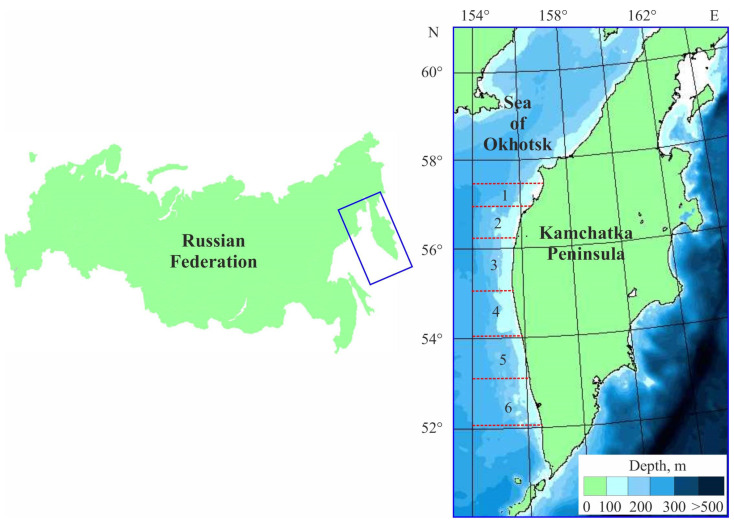
Map of the Sea of Okhotsk with subdivision of the western Kamchatka shelf into red king fishery areas. 1—Khairyuzovskiy Area; 2—Northern Closed Area; 3—Ichinskiy Area; 4—Kolpakovskiy Area; 5—Kikhchikskiy Area; and 6—Ozernovskiy Area (adopted from [5]).

The western Kamchatka shelf hosts the most significant fishing area for *P. camtschaticus*, with crabs forming commercial density concentrations at depths ranging from 50 to 200 m. This area is divided into six zones in accordance with stock concentrations and migration patterns of red king crabs [75]: (a) the Khairyuzovskiy Area (57°00′ to 57°30′ N); (b) the Northern Closed Area (56°20′ to 57°00′ N); (c) the Ichinskiy Area (55°10′ to 56°20′ N); (d) the Kolpakovskiy Area (54°00′ to 55°10′ N); (e) the Kikhchikskiy Area (53°00′ to 54°00′ N); and (f) the Ozernovskiy Area (51°00′ N to 53°00′ N) (Figure 1).

The red king crab fishery began in the Ichinskiy Area in the early 1920s, before later becoming primarily concentrated in northern areas such as the Ichinskiy Area and, particularly, the Khairyuzovskiy Area. During periods of high population abundance, fisheries were present only in the Kikhchikskiy and Ozernovskiy Areas.

Large-scale fisheries commenced in 1925, with annual landings reaching 30 million crabs by 1927. These elevated catch rates contributed to a significant decrease in the overall red king crab population. Annual landings between 1932 and 1935 decreased to 14.0–21.5 million individuals. The abundance of *P. camtschaticus* sharply declined in southern areas, such as the Ozernovskiy Area, where fishermen harvested no crabs, and in the Kikhchikskiy Area, where the fishery targeted juveniles and male crabs with CW < 150 mm [76]. In 1937 and 1938, red king crab landings returned to 30 million crabs per year [72]. However, during World War II, king crab harvests along west Kamchatka nearly ceased. The gradual growth in catch efforts after the war resulted in a sharp increase in abundance across all subpopulations, particularly between 1946 and 1949. Combined Russian and Japanese landings reached 29 million crabs in 1956 and 1957 [72]. Due to the high importance of the northern shelf for the recruitment of *P. camtschaticus*, the Northern Closed Area was established in 1959 to protect nursery grounds and areas of crab propagation [63]. During the years 1964 to 1968, the annual landings of legal male crabs (CW > 150 mm) were considerably high, ranging between 25.5 and 28.3 million. Following this period, a decline in the annual catches was observed, decreasing from 24 million crabs in 1969 to 14.5 million crabs in 1974. In 1975, the Russian fleet shifted to harvesting red king crabs using pots, which led to relatively low landings between 1975 and 1981, with an average of 8.5 million crabs caught annually. In the period from 1996 to 2000, Russia became the dominant player in global king crab fisheries, contributing approximately 80–90% of the total supply [77]. It is noteworthy that the reported catches of 23,600 to 37,700 tons were significantly lower compared to the actual values of 56,000 to 59,400 tons [5]. The high catch rates led to a substantial decline in the abundance of commercial males in the southern areas, where fishing was most intensive, ranging from two to seven times [75]. A further decline in stock indices occurred during the 2001 to 2004 period, when reported catches gradually decreased from 16 to 2 thousand metric tons [78]. The western Kamchatka shelf fishery was reopened in 2007, with a total allowable catch (TAC) of 3927 tons. That same year, following a two-year ban, the official annual catch for red king crabs increased to 4700 tons. Consequently, the abundance indices declined significantly, leading to the closure of the fishery. During the fishery closure period, the TACs were limited to 0.1 to 1300 tons (allocated for research purposes), although the actual catches were much higher, ranging between 10,000 and 18,000 tons [78]. The commercial fishery of red king crab in the western Kamchatka waters resumed in 2013, with a total annual catch of 5520 tons. In 2014, this figure dropped to 4950 tons but experienced an increase over the next three years, fluctuating between 7210 and 11,800 tons [78]. In 2018 and 2019, the annual catch remained comparatively stable, maintaining a consistent yield of approximately 15,300 tons [79]. In subsequent years, the total annual catch exhibited a modest decline, reaching approximately 14,000 tons in 2024.

## 4. Species Composition of Symbionts

An examination of the symbiotic species associated with red king crabs in the Sea of Okhotsk revealed a diverse assemblage of forty-two taxa (Table 1), with ciliates displaying the highest species richness (11 species), followed by crustaceans (8 species) and acanthocephalans (4 species).

Prevalent and abundant symbiotic associates include ciliate protozoans such as *Lagenophrys* sp. *Acineta tuberosa*, *Epistylis* sp., and *Zoothamnium* sp., the kamptozoan Loxosomatidae gen sp., the barnacle *Hesperibalanus hesperius*, the fish leech *Johanssonia arctica*, and the amphipod *Ischyrocerus commensalis* [58,59,60].

**Figure 2:**
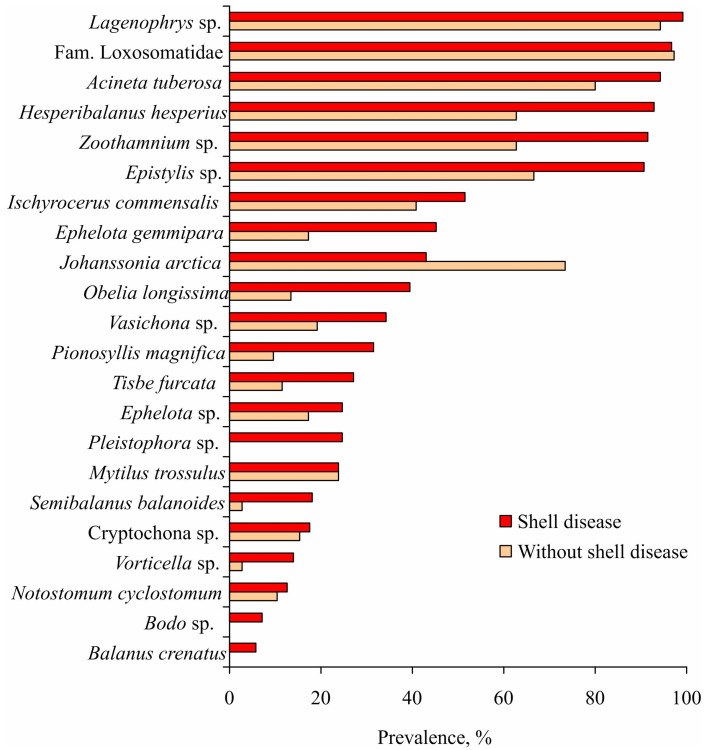
Prevalence of symbionts on shell disease and healthy red king crabs in the Sea of Okhotsk (adopted from [60]).

Approximately 10–11% of red king crabs in the study population exhibited shell disease, a condition induced by bacterial agents including *Vibrio alginolyticus*, *V. alginolyticus*, *V. vulnificus* and *V. fluvialis* [58,59,80,81,82] as well as *Aeromonas*, *Pseudomonas*, *Alteromonas*, and *Flavobacterium* [83]. The symbiotic community structure of afflicted crabs was markedly distinct from that of healthy individuals, exhibiting greater species diversity (42 vs. 21 species) and elevated prevalence levels for the majority of symbiotic taxa (Figure 2).

## 5. Localization Patterns of Symbionts and Their Relationships with the Host

### 5.1. Parasites

In the Sea of Okhotsk, parasitic associations were observed exclusively among crabs suffering from shell disease, with infection levels remaining relatively low. The heterotrophic flagellate *Bodo* sp. (annual prevalence: 6.7–12.0%) and the ciliate *Mesanophrys* sp. (prevalence: 1.7–7.5%) were found to infect their hosts’ hemolymph and gills. In contrast, the dinoflagellate *Hematodinium* sp. and the amoeba *Paramoeba perniciosa* (prevalence: 2%) infected only the hemolymph, while the ciliate *Chilodonella* sp. (prevalence: 2.0–7.7%) colonized the gills of red king crabs.

High densities of *Bodo* sp. can lead to gill and surface fouling disease in crustacean hosts, particularly when the host is under stress [84]. Scuticociliates of the genus *Mesanophrys* have been reported to cause significant pathologies in their hosts, including severe morbidity, mortality, and, in some instances, castration [85]. The crab species *Carcinus maenas*, *Cancer pagurus*, *Metacarcinus magister*, and *Callinectes sapidus* have been documented as hosts for four species of scuticociliates [38,86].

**Figure 3:**
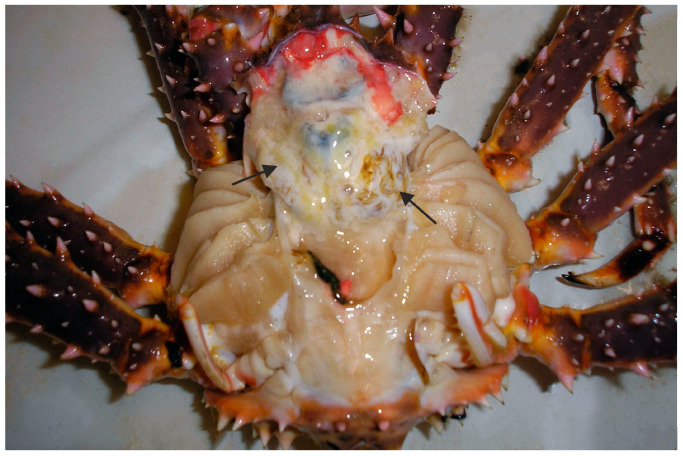
Red king crab infected with the dinoflagellate *Hematodinium* sp. Arrows show infected tissues (modified from [54]).

*Hematodinium* sp. or *Hematodinium*-like infections have been documented in various cold-water crustacean species, such as the Norway lobster (*Nephrops norvegicus*), oregonid crabs (*Chionoecetes* spp.), and lithodid crabs [54,57,87,88,89]. *Hematodinium perezi* has also been identified in portunid, polybiid, and cancrid crabs from North America, Europe, and China [38,86,90,91,92,93,94]. Patently infected crabs often display lethargy when infected by *Hematodinium* spp., developing a condition known as bitter crab disease (BCD). BCD-afflicted crabs exhibit milky white hemolymph (Figure 3) due to high parasite densities, with parasites commonly infiltrating host connective tissue, obstructing hemolymph sinuses and blood-carrying channels, often resulting in dilation [95].

Organ epithelia may become attenuated, highly vacuolated, and necrotic [54]. In the Sea of Okhotsk, there is a trend of decreasing prevalence of this parasite from the south to the north of the area [61]. A recent study by Ryazanova et al. [95] reported that the prevalence rate of this parasite in red king crabs near the Pacific coast of Kamchatka (Pacific Ocean) was 2.7%, which is higher than in the Sea of Okhotsk.

**Figure 4:**
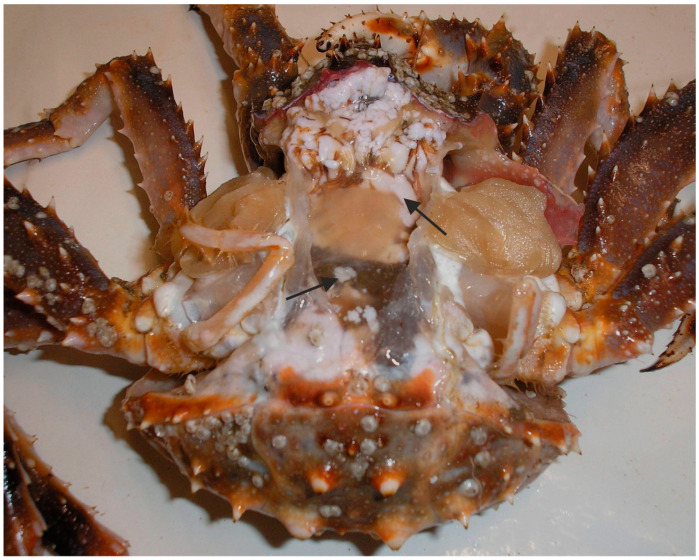
Red king crab infected with the microsporidian *Thelohania* sp. Arrows show infected tissues (adopted from [57,60]).

The microsporidian *Pleistophora* sp. has been identified as a common parasite of red king crabs, with prevalence levels ranging from 12% to 50%. The parasite localizes in the hepatopancreas, antennal gland, digestive tract, and muscles of the host. Another microsporidian species, *Thelohania* sp., has been found in the external organs and muscles of red king crabs (Figure 4).

Microsporidia are obligate intracellular parasites lacking mitochondria, with spores possessing a single polar filament—a specialized organelle facilitating host invasion. In severe cases, microsporidian infections in skeletal muscle may result in white, opaque muscle fibers observable through the semi-transparent chitin covering of the king crab abdomen, initiating the development of melanized lesions in the tissues [55]. *Pleistophora cargoi* and a closely related species have been documented in the muscles of the blue crab (*Callinectes sapidus*) [96], while *Pleistophora crangoni* has been reported as a parasite of *Crangon franciscorum*, *C. nigricauda*, and *C. stylirostris* in Yaquina Bay, Oregon [97].

Analysis of red king crabs revealed two species of nematodes with low prevalence and abundance levels in the digestive tract. Specifically, *Anisakis* sp. l. and *Hysterothylacium aduncum* exhibit prevalence ranges of 1.7–7.7% but occur in low abundances of just one to two specimens. While occasional reports document the existence of *Anisakis* sp. larvae in the visceral region of the great spider crab, *Hyas araneus*, from the coastal waters of the Barents Sea, prevalence is noted to be very low at just 0.12% [98]. Further, larval stages of *Anisakis* sp. have been detected in the viscera of *Cancer plebejus* in Chilean waters [99].

Additional analyses detected four species of acanthocephalans (*Corynosoma strumosum*, *Bolbosoma caenoforme*, *Polymorphus botulus*, and *Echinorhynchus gadi*) in the stomachs of the crabs. Prevalence levels were relatively low, ranging from 2.0 to 3.3%, while intensity was noted to be one to two specimens per crab [100,101]. Apart from these findings, occasional instances of acanthocephalan parasites at their larval stages have been reported in red king crabs from the Barents Sea [100,101]. In terms of the propagation of acanthocephalans, the intermediate host usually gets infected when it consumes eggs containing an acanthor; this occurs passively without any active involvement on the part of the host. The acanthor departs from the egg and migrates through the host’s digestive tract, eventually settling in the hemocoel or serosa. The acanthella, which results from the continued development of the acanthor, leads to the formation of the cystacanth—the stage that culminates in infectivity to the final host. Infection by larval acanthocephalan results in local cell and tissue necrosis during migration and enlargement phases, characterized by compression atrophy and disorganization of surrounding tissues [38].

Small unidentified turbellarians were found in the gills of several red king crabs from the Sea of Okhotsk. For comparison, commensal turbellarians belonging to the genus *Ectocotyla* have been reported on the gills and branchial chambers of brachyuran crabs from the east coast of Canada [102].

In the Sea of Okhotsk, 2.5% of all red king crabs were infected by the rhizocephalan barnacle *Briarosaccus callosus* [60]. Adult females of this species are sedentary, consisting of an externa, which exhibits a sac-like appearance, and an interna, which serves as the nutrient-absorbing component of the parasite and ramifies throughout the host’s body. Males are reduced to spermatogenic cells that reside within the male receptacles of the externa [103]. Rhizocephalans have a considerable impact on the host’s physiology, morphology, and behavior. All organs of the host, including the gonad, hepatopancreas, muscle, and neural tissue, as well as the eyestalk, androgenic gland, and thoracic ganglion, are invaded. The invasion of the eye, androgenic gland, and thoracic ganglion causes significant alterations in the host’s neurosecretory functions, affecting molting and endocrine functions. The invasion of the gonad results in host sterilization, with infected males becoming feminized through successive molts and acquiring female secondary sexual characteristics [103,104]. In the North Pacific, *Briarosaccus callosus* is more frequently documented as a parasite of deep-water crab species such as *Paralithodes platypus*, *Lithodes couesi*, *L. aequispina*, *Paralomis multispina*, and *P. verrilli* [105]. The parasite also infects other lithodid crabs globally. Recently, two new species of *Briarosaccus* have been distinguished using genetic markers (COI and 16S). One such species, *B. auratum*, was discovered on the golden king crab *Lithodes aequispinus*, while the second undescribed species parasitized *Paralithodes camtschaticus* and *P. platypus* [106]. Therefore, the prevalence rates for all parasites were found to be relatively low, and these organisms were identified exclusively in crabs affected by shell disease. This finding suggests that the red king crab population in the Sea of Okhotsk is healthy, a conclusion that is further substantiated by the high annual catch rates (see above).

### 5.2. Microepibionts

**Figure 5:**
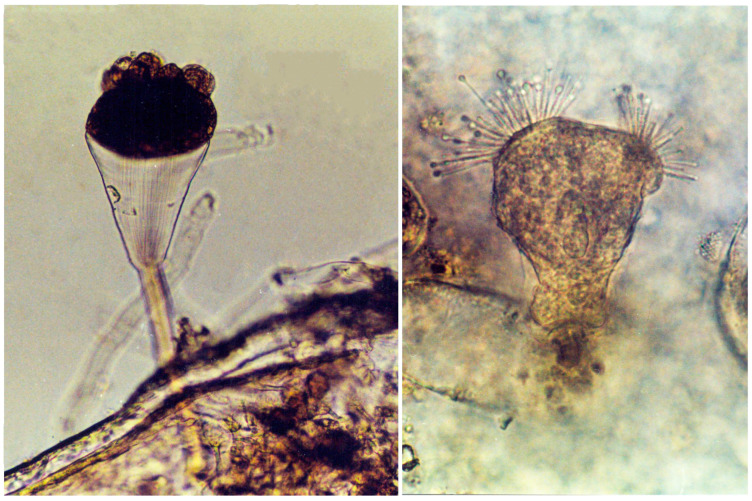
Suctorians *Ephelota* sp. (**left**) and *Acineta tuberosa* (**right**) on the gills of a red king crab from the Sea of Okhotsk. × 100 magnification (modified from [60]).

Suctorian ciliates, including *Ephelota gemmipara*, *Ephelota* sp., and *Acineta tuberosa*, and peritrichous ciliates *Epistylis* sp., *Zoothamnium* sp., and *Lagenophrys* sp. typically infest the gills of red king crabs (Figure 5) at a high rate, with infestation indices ranging from 50 to 100% (prevalence) and 13.2 to 73.65 individuals per crab (mean intensity).

Ciliates were also found on the eggs of female *P. camtschaticus*. Other peritrichous ciliates, such as *Apiosoma* sp. and *Vorticella* sp., demonstrated similar localization patterns. The chonotrich ciliate *Vasichona* sp. was identified on the mouthpart setae (prevalence 16–69%). The same localization, but with lower infestation indices, was reported for the chonotrich ciliate *Cryptochona* sp. [60]. Ciliates are known to be prevalent epibionts of decapod crustaceans in both marine and freshwater environments [24,107]. *Ephelota* sp. was registered on the gills of the lobster *Homarus americanus* and crabs *Cancer irroratus*, *C. borealis*, *Ovalipes ocellatus*, and *Callinectes sapidus* in Sandy Hook Bay, New Jersey, and at near-shore stations in New York [108]. *Ephelota plana* and *Zoothamnium* sp. were documented on the chelipeds, carapace, and anterior pereiopods of the crabs *Liocarcinus depurator* and *Pilumnus hirtellus*, collected on the west coast of Scotland [109]. The suctorians *Ephelota plana*, *Acineta compressa*, *Conchacineta constricta*, and *Corynophrya anisostyla*, the peritrichs *Cothurnia mobiusi* and *Zoothamnium plumula*, and the chonotrich ciliate *Chilodochona quennerstedti* were found on the hermit crab *Pagurus bernhardus* from Scotland [109]. Juvenile southern king crabs (*Lithodes santolla*) harbored a diverse community of epibionts, including the following ciliate species: *Ephelota gemmipara*, *Ephelota gigantea*, *Podophrya fixa*, *Acineta tuberosa*, *Zoothamnium duplicatum*, *Chilodochona quennerstedti*, and *Gymnodinioides* sp. [110]. Epibiotic ciliates appear to have no adverse effects on their decapod hosts, although certain drawbacks related to heavy gill fouling could be expected.

### 5.3. Mesoepibionts

This group was represented by a single organism, a member of the family Loxosomatidae belonging to Entoprocta or Kamptozoa. It was abundant on the surface of red king crab gills, with an annual prevalence/mean intensity ranging from 83.3 to 100%/12.0–35.5 individuals per gill filament in crabs with shell disease and 70.0–100%/4.2–35.4 individuals per gill filament in crabs without shell disease [60]. These small (0.2–8 mm), sessile or slow-moving solitary invertebrates typically live as epibionts of various types of other invertebrates, including polychaetes, bryozoans, and sponges, among others [111].

### 5.4. Macropibionts

**Figure 6:**
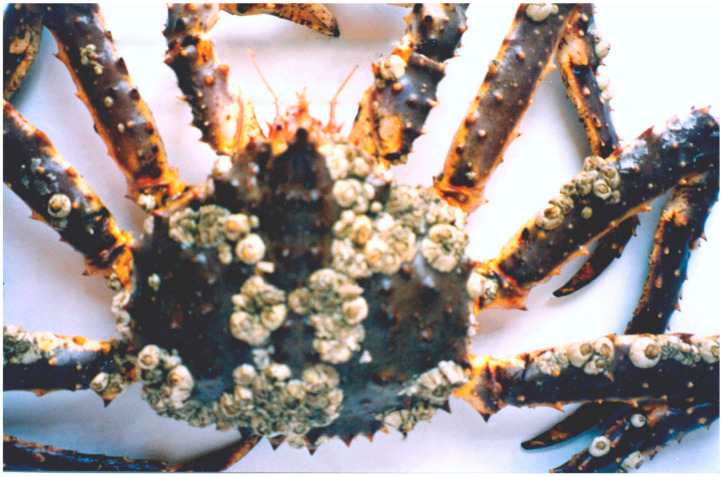
Barnacles *Hesperibalanus hesperius* and *Semibalanus balanoides* on the carapace and limbs of a red king crab from the Sea of Okhotsk (modified from [60]).

The most commonly observed macroepibionts were the barnacles *Hesperibalanus hesperius* (with an annual prevalence of 78–100% and a mean intensity of 58.9–108.3 individuals per crab), *Semibalanus balanoides* (3.8–37.5%, 1.6–5.7 individuals per crab), and *Balanus crenatus* (2.0–23.1%, 1.0–16.5 individuals per crab), which were found on the carapace (Figure 6).

The same localization was observed for *Chirona evermanni* [60]. Barnacles are sessile animals with no specific substrate preferences, so it is not surprising that they colonize both dead and living substrates, including the hard body parts of crustaceans [37]. The two barnacle species (*Semibalanus balanoides* and *Balanus crenatus*), found on red king crabs from the Sea of Okhotsk, were also recorded on the carapace and limbs of the same host in the Barents Sea, along with *Balanus balanus* [44,46,47,48]. Barnacle infestation indices were higher in the Sea of Okhotsk, likely due to a larger proportion of crabs with older exoskeletons. When barnacles occupy the shell of a crustacean host in significant numbers, they may add weight to the host’s shell [112,113], potentially resulting in negative effects such as decreased mobility and increased energy consumption. In the case of the red king crab, a species known for its large body and weight, such effects are unlikely [46,47,48].

Free-living amphipods *Ischyrocerus commensalis* (15.4–88.0%, 6.0–14.6 individuals per crab) and harpacticoid copepods *Tisbe furcata* (10.0–76.9%, 1.3–4.7) were found inhabiting the gills of red king crabs from the Sea of Okhotsk. In contrast, the amphipod *Caprella ungulina* was found under the abdomen, with a prevalence of 12.0–53.9% and a mean intensity of 1.3–10.4 individuals per crab [60]. Amphipods of the genus *Ischyrocerus* are known symbionts of the Tanner crab, *Chionoecetes bairdi*, found around Kodiak Island [114], and the snow crab, *Chionoecetes opilio*, from Bonne Bay, Newfoundland [115]. *Ischyrocerus commensalis* and its congener, *I. anguipes*, are also common symbionts of shelf crab species in the Barents Sea [116,117,118,119,120,121,122]. However, there are significant differences in infestation indices and localization between these organisms. Indeed, in the Barents Sea, amphipods of the genus *Ischyrocerus* are rather abundant on the mouthparts and limbs of red king crabs, with prevalences and mean intensities higher than those found on crabs from the Pacific region [46,47,121]. Furthermore, small numbers of these amphipods were recorded on the eggs of red king crabs, but laboratory observations refuted the hypothesis of egg predation [120]. Nevertheless, negative effects may arise from symbiotic amphipods when they are concentrated in a host’s gills, possibly leading to impaired respiration and increased energetic costs associated with cleaning activities of the fifth pair of rudimentary walking legs [117]. The copepod *Tisbe furcata* and other harpacticoids are well-known symbionts of red king crabs in the Barents Sea [46,47,123,124,125]. They also prefer to inhabit the host’s gills, but infestation intensities in the Barents Sea are 5–10 times higher than in the Sea of Okhotsk. Consequently, these copepods may have the same negative impact on the host as symbiotic amphipods.

**Figure 7:**
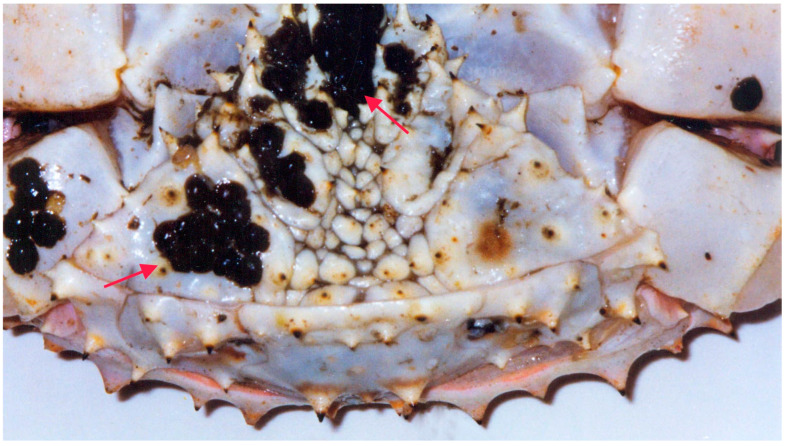
Cocoons of the fish leech *Notostomum cyclostomum* (arrows) on the abdomen of a red king crab from the Sea of Okhotsk (modified from [60]).

In the Sea of Okhotsk, fish leeches exhibited the highest infestation prevalence and mean intensity, ranging as follows: *Johanssonia arctica* (23.3–76.9%; 2.0–4.2 individuals per crab), *Notostomum cyclostomum* (4.0–24.0%, 1.0–3.1 individuals per crab), and *Crangonobdella maculosa* (4.0–16.7%, 1.0–2.3 individuals per crab). These three species exhibited a preference for colonizing the carapace and limbs. Cocoons of *J. arctica* and *C. maculosa* were more frequently observed under the abdomen and on the limbs, while *N. cyclostomum* cocoons were typically found on both the internal and external surfaces of the exoskeleton (Figure 7).

*Johanssonia arctica* was also recorded on red king crabs in the Barents Sea with the same localization, albeit with lower infestation indices [44,46,47,126]. Fish leeches are commensals that utilize their crustacean hosts for egg deposition [127].

**Figure 8:**
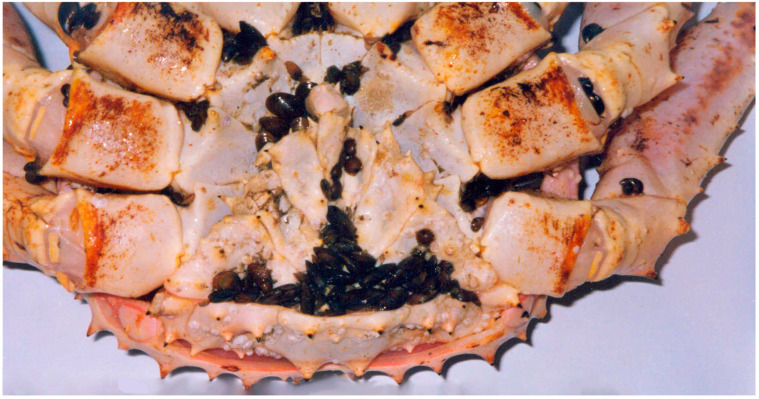
Bivalve mollusk *Mytilus trossulus* on the abdomen of a red king crab from the Sea of Okhotsk (modified from [60]).

The bivalve mollusk *Mytilus trossulus* attached itself to the ventral portion of the carapace, abdomen (Figure 8), limbs, and, less frequently, gills.

Annual prevalence ranged from 10.0 to 53.9%, while mean intensity varied between 1.9 and 25.2 individuals per crab. The mollusk *Arvella manschurica* exhibited the same localization, except for the gills, with lower infestation indices (5.0–7.7%, 1–2 individuals per crab) [60]. Mytilids are well-known epibionts of various crustacean hosts [37,128].

**Figure 9:**
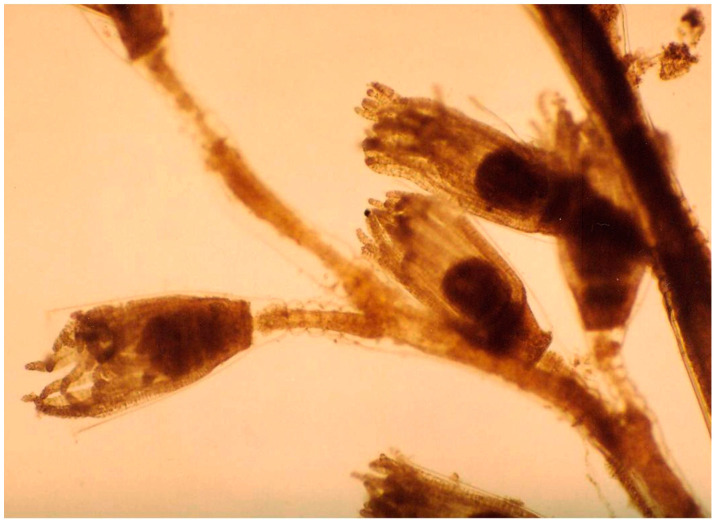
Hydrozoan *Obelia longissima* on the carapace of a red king crab (modified from [60]).

Hydrozoans *Obelia longissima* and *Sertularia cupressoides* typically colonized the carapace (Figure 9) and limbs with annual prevalences of 20.0–52.5% and 11.7%, respectively [60].

*Obelia* spp. have been found in association with a wide range of crustacean hosts, including hermit crabs, snow crabs [37,129], and lithodid crabs from the Barents Sea [25,46,47,116,122]. Hydrozoans associated with red king crabs in the Sea of Okhotsk represent the only epibionts that do not cause detrimental effects to their hosts.

The spirorbid polychaete *Circeis armoricana* was found attached to the carapace with annual infestation indices of 2.0–7.7% and a range of 1.5–9.7 individuals per crab, whereas the mobile, free-living worms *Pionosyllis magnifica* (5.0–47.5%, 1.4–10.0 individuals per crab) and *Exogon gemmifera* (2.5–4.0%, 1 individual per crab) were observed in the branchial chambers of crabs from the Sea of Okhotsk [60]. Spirorbids are known as non-specific fouling organisms found on a wide range of hosts, from small hermit crabs [37] to large, commercially important crab species [44,46]. In the Barents Sea, *Circeis armoricana* colonizes red king crabs to a greater extent than in the Sea of Okhotsk [46,47]. The impacts of mobile, free-living polychaetes on host red king crabs are debated. On one hand, their infestation indices are relatively low for the majority of red king crabs, yet in cases of high gill intensity, they may impair host respiration.

## 6. Implications for Fishery Management

Symbionts have been identified as significant contributors to declines in crab populations [43,130,131]. For instance, *Hematodinium perezi*, which prefers high-salinity waters [90], is prevalent in blue crabs (*Callinectes sapidus*) in the United States and is believed to greatly impact their populations [132]. In years characterized by high prevalence, this agent has resulted in reduced catches and increased proportions of lethargic, moribund, and dead blue crabs in pots [90]. Notably, mortalities of this host (30% per month) have been recorded during epizootics induced by *Paramoeba perniciosa* [133]. Viral and microsporidian parasites have been implicated in the decline of Bristol Bay’s red king crab population abundance [43].

Nemertean worms from the genus *Carcinonemertes*, notorious egg predators across various crab species, can lead to the total loss of an egg clutch, a condition akin to parasitic castration, resulting in declines in certain fishery stocks [134,135]. For example, the influence of *Carcinonemertes errans* on the population density of *Cancer magister* has been demonstrated to be comparable to density-dependent recruitment mechanisms [136]. The red king crab population in Bristol Bay has also experienced parasite-associated brood mortality due to nemerteans [135].

Rhizocephalans, among other parasites, are the most recognizable in relation to king crab infestations and may potentially have the most significant impact on these populations. Consequently, in areas with a high prevalence of *Briarosaccus callosus*, king crabs are generally smaller, and sex ratios can become skewed due to parasitic castration [43].

Therefore, extensive disease research and surveys of other symbionts are necessary to obtain reliable pre-epizootic data for the sustainable management and investigation of crab stocks. Nonetheless, available data on red king crab symbionts in the Sea of Okhotsk suggest that hazardous crab-associated organisms, such as microsporidians and rhizocephalans, exhibit a low infestation prevalence, while egg predators are absent. Consequently, acute diseases are unlikely to have a chronic impact on the distribution and population abundance of red king crabs in the Sea of Okhotsk. This assertion is corroborated by positive trends in commercial stocks and annual landings of this species in this region.

## 7. Conclusions

In the Sea of Okhotsk, at least 42 symbiotic species can be identified in association with the red king crab. These parasites include heterotrophic flagellates, such as *Bodo* sp., ciliates like *Mesanophrys* sp. and *Chilodonella* sp., dinoflagellates, such as *Hematodinium* sp., amoebae, such as *Paramoeba perniciosa*, microsporidians like *Pleistophora* sp., nematodes, such as *Anisakis* sp., acanthocephalans, including *Corynosoma strumosum*, *Bolbosoma caenoforme*, *Polymorphus botulus*, and *Echinorhynchus gadi*, one unidentified turbellarian worm, and the rhizocephalan barnacle *Briarosaccus callosus*. Among these parasitic organisms, ciliates were the most abundant. Crustaceans constituted the majority of macroepibionts, followed by fish leeches, bivalve mollusks, hydrozoans, and polychaetes. Although certain species, such as rhizocephalan and *Hematodinium*-like hemolymph parasites, pose a serious threat to the health of the crab and could potentially lead to significant declines in population abundance, their prevalence among red king crabs in the Sea of Okhotsk is low, and, at present, they do not cause issues for the local fishery.

## Figures and Tables

**Table 1:** List of symbionts found on red king crabs from the Sea of Okhotsk (modified from [60]).

Taxa	Prevalence, %	Intensity, ind.	
		Range	Mean
**Protozoa (Euglenozoa)**			
*Bodo* sp.	5.5	–	–
**Protozoa (Amoebozoa)**			
*Paramoeba perniciosa*	0.2	–	–
**Dinoflagellata**			
*Hematodinium* sp.	1.8	–	–
**Ciliophora**			
*Acineta tuberosa*	90.8	1–128	19.9
*Apiosoma* sp.	3.7	1–10	2.3
*Chilodonella* sp.	0.2	–	–
*Cryptochona* sp.	16.9	1–85	4.0
*Ephelota gemmipara*	38.4	1–62	19.2
*Ephelota* sp.	22.9	1–28	3.3
*Epistylis* sp.	84.9	1–101	15.0
*Lagenophrys* sp.	97.9	1–181	33.4
*Vasichona* sp.	30.7	1–120	8.5
*Vorticella* sp.	11.2	1–67	3.6
*Zoothamnium* sp.	84.7	1–155	20.1
**Fungi (Microsporidia)**			
*Pleistophora* sp.	18.8	–	–
*Thelohania* sp.	0.2	–	–
**Entoprocta**			
Fam. Loxosomatidae	96.8	1–87	19.3
**Hydrozoa**			
*Obelia longissima*	33.2	1–200	23.2
*Sertularia cupressoides*	0.5	1–9	4.0
**Turbellaria**			
Turbellaria gen sp.	0.7	1–1	1.0
**Nematoda**			
*Anisakis* sp., larva	1.6	1–2	1.3
*Hysterothylacium aduncum*, larva	1.6	1–2	1.3
**Acanthocephala**			
*Corynosoma strumosum*, juv.	0.5	1–1	1.0
*Bolbosoma caenoforme*, iuv.	1.4	1–3	2.0
*Echinorhynchus gadi*, iuv.	0.5	2–2	2.0
*Polymorphus botulus*, juv.	0.9	1–2	1.3
**Polychaeta**			
*Pionosyllis magnifica*	26.3	1–17	2.4
*Circeis armoricana*	2.3	1–20	5.2
*Exogone gemmifera*	1.4	1–1	1.0
**Hirudinea**			
*Johanssonia arctica*, adult	50.3	1–31	4.0
*Johanssonia arctica*, cocoons	68.4	1–990	111.6
*Notostomum cyclostomum*, adult	12.1	1–8	2.3
*Notostomum cyclostomum*, cocoons	31.4	1–55	8.4
*Crangonobdella maculosa*, adult	2.5	1–5	2.1
*Crangonobdella maculosa*, cocoons	10.3	1–20	3.3
**Mollusca**			
*Arvella manshurica*	3.0	1–3	1.3
*Mytilus trossulus*	23.8	1–110	12.1
**Crustacea**			
*Briarosaccus callosus*	0.5	1–1	1.0
*Tisbe furcata*	23.3	1–20	2.7
*Balanus crenatus*	4.3	1–23	4.6
*Chirona evermanni*	0.5	1–1	1.0
*Hesperibalanus hesperius*	85.6	1–1000	81.1
*Semibalanus balanoides*	14.4	1–11	3.2
*Caprella ungulina*	3.0	1–30	6.2
*Ischyrocerus commensalis*	49.0	1–40	9.9

## Data Availability

No new data were created or analyzed in this study. Data sharing is not applicable to this article.
